# Novel Protein-Protein Interactions Inferred from Literature Context

**DOI:** 10.1371/journal.pone.0007894

**Published:** 2009-11-18

**Authors:** Herman H. H. B. M. van Haagen, Peter A. C. 't Hoen, Alessandro Botelho Bovo, Antoine de Morrée, Erik M. van Mulligen, Christine Chichester, Jan A. Kors, Johan T. den Dunnen, Gert-Jan B. van Ommen, Silvère M. van der Maarel, Vinícius Medina Kern, Barend Mons, Martijn J. Schuemie

**Affiliations:** 1 Biosemantics Association, Department of Human Genetics, Leiden University Medical Center, Leiden, and Department of Medical Informatics, Erasmus University Medical Center, Rotterdam, The Netherlands; 2 Post-Graduate Program in Knowledge Engineering and Management (EGC), Federal University of Santa Catarina (UFSC), Florianópolis, Brazil; Science Commons, United States of America

## Abstract

We have developed a method that predicts Protein-Protein Interactions (PPIs) based on the similarity of the context in which proteins appear in literature. This method outperforms previously developed PPI prediction algorithms that rely on the conjunction of two protein names in MEDLINE abstracts. We show significant increases in coverage (76% versus 32%) and sensitivity (66% versus 41% at a specificity of 95%) for the prediction of PPIs currently archived in 6 PPI databases. A retrospective analysis shows that PPIs can efficiently be predicted before they enter PPI databases and before their interaction is explicitly described in the literature. The practical value of the method for discovery of novel PPIs is illustrated by the experimental confirmation of the inferred physical interaction between CAPN3 and PARVB, which was based on frequent co-occurrence of both proteins with concepts like Z-disc, dysferlin, and alpha-actinin. The relationships between proteins predicted by our method are broader than PPIs, and include proteins in the same complex or pathway. Dependent on the type of relationships deemed useful, the precision of our method can be as high as 90%. The full set of predicted interactions is available in a downloadable matrix and through the webtool Nermal, which lists the most likely interaction partners for a given protein. Our framework can be used for prioritizing potential interaction partners, hitherto undiscovered, for follow-up studies and to aid the generation of accurate protein interaction maps.

## Introduction

Protein-protein interactions (PPIs), which we define as proteins that physically interact, are crucial in most complex biological processes. Experimental high-throughput methods such as yeast two-hybrid screens have been used to make large inventories of PPIs and to create protein interaction maps [1]–[6]. However, it is well known that these methods merely show physical interaction under experimental condition and not necessarily indicate a common involvement in a biological process. Computational methods for the prediction of PPIs could theoretically aid the discovery of candidate biological interaction partners. There are many different sources of information that can be used in PPI prediction [7], including protein structures, phylogenetic distribution, interactions between homologous proteins in other organisms, genomic neighborhood, and gene fusions. In this article, we will focus on one source of information, which is arguably the most comprehensive, but also the least structured: biomedical literature itself. Until now text mining techniques are mainly used to rediscover PPIs explicitly described in literature. Often, the now 18 million freely available abstract records of MEDLINE are used for this purpose. PPIs extracted this way have been shown to improve the accuracy of predicted biological networks [8], [9]. Structured information on explicit PPIs extracted from MEDLINE and other sources is freely available in the STRING database [10], or can be found by querying the iHOP website [11].

However, text mining can go one step further; by combining known associations, previously unknown PPIs can be inferred. Because most text mining research, including this study, limits itself to MEDLINE abstracts, these ‘previously unknown’ interactions also include interactions that are effectively known, but not explicit in MEDLINE as they are only mentioned in a full text article. Swanson [12], [13] *et al.* were the first to demonstrate that text mining can lead to the discovery of new knowledge (e.g. the treatment of Raynaud's disease by fish oil). Other studies in the biomedical domain verified the importance of implicit information for knowledge discovery [14]–[16]. Whereas Swanson used a word-based approach, linking entities by intermediate words that appeared frequently in the contexts of both entities, in our work we use a concept-based approach: different terms denoting the same concept (*i.e.* synonyms) are mapped to a single concept identifier, and ambiguous terms, e.g., identical terms used to indicate different concepts (*i.e.* homonyms) are resolved by a disambiguation algorithm. Such an approach is essential given the wide diversity and many ambiguities in gene and protein nomenclature [17], [18].

In order to predict PPIs, we summarize the typical context in which each protein appears into *concept profiles* [15], [16], [19]. We hypothesize that a high similarity between the concept profiles of two proteins is indicative for an actual biological interaction. For example, if two proteins are consistently mentioned together with a particular disease, the probability that these proteins interact is higher than the a priori probability of two randomly selected proteins [20], [21]. This probability should increase further when they are also frequently co-mentioned with a particular pathway, a sub-cellular localization, or other proteins.

In this article, we first demonstrate the added value of a concept-based approach over a traditional term-based approach in detecting explicitly described relations. We proceed to show the added value of the concept profile-based approach over classical direct relation extraction, including the text-mining techniques used in the STRING database. Subsequently, we show the predictive power of our method by doing a retrospective study; we demonstrate that we can employ the literature available in 2005 to predict 52% of the PPIs newly described in Swiss-Prot in 2007 at a specificity level of 95%. We show that in addition, some of the PPIs that we predicted but are not yet recorded in any database represent indirect protein interactions and have biological relevance. Finally, we confirm one of the many predicted PPIs in three wet lab experiments, supporting our claim that the concept profiling method is capable of previously unknown PPI prediction from current literature.

These predictions will be useful for (i) the ranking of potential PPIs for more specific experimental analysis, and (ii) complementing other types of data such as co-expression and yeast two-hybrid data when using an integrative systems biology approach.

## Results

### Improved PPI Detection Using Concept Profiles

We compared the performance of different PPI prediction approaches in detecting known human PPIs in MEDLINE. The online human-curated databases Biogrid, DIP, HPRD, MINT, Reactome, and UniProt/Swiss-Prot were used to establish a set of 61,807 known human PPIs. A set of probable Non-Interacting Protein Pairs (NIPPs) was generated from all pairs of proteins that do not occur in the above databases nor in the IntAct [22] database, which includes, in addition to all PPIs recorded in UniProt/Swiss-Prot, many non-curated PPIs from high-throughput experiments. We compare four approaches:

- *Word-based direct relation*. This approach uses direct PubMed queries (words) to detect if proteins co-occur in the same abstract. This is the simplest approach and represents how biologists might use PubMed to search for information.
- *Concept-based direct relation*. This approach uses concept-recognition software to find PPIs, taking synonyms into account, and resolving homonyms. Here two concepts (in our case two proteins) are detected if they co-occur in the same abstract.
- *STRING* [*10*]. The STRING database contains a text mining score which is based on direct co-occurrences in literature.
- *Concept profile-based relation*. This approach uses the similarity in literature context. Here two proteins (concepts) can also be indirectly related via the concepts in their profiles. More detail on concept profiles and their construction can be found in the Methods section.

The word-based and concept-based direct relation methods could find at least one abstract containing both proteins for respectively 33% and 32% of the pairs in the PPI set. A text mining score from STRING could be obtained for 30% of the PPIs, in line with the co-occurrence based approach used to create STRING. Thus, a majority of the known PPIs cannot be found explicitly in MEDLINE. For the concept profile-based approach, we could create concept profiles and calculate a similarity score for 76% of the PPI set.

Similar to STRING, the other three approaches can also be used to calculate a continuous score that indicates the strength of the relation between two proteins. Figure S1 displays the distribution of the similarity scores of the concept profile-based method for the PPI and NIPP sets. This figure shows that the scores for the PPI set are higher although there is also overlap between the two distributions. The continuous scores can be used to rank protein pairs. After ranking the pairs in the PPI and in the NIPP set, we calculated the sensitivity at a specificity of 99% and 95%, and the Area under the Curve (AuC), which is often used in the evaluation of classifiers, and expresses the area under the Receiver Operator Characteristics (ROC) curve (see supplement S5 in Supporting Information File S1). An AuC of 0.5 indicates a random classifier; an AuC of 1 indicates a perfect classifier. For this analysis, we limited ourselves to those pairs in the PPI and NIPP set for which all methods could make a prediction. We analyzed 44,920 pairs in the PPI set, and 58,388,409 pairs in the NIPP set.

The results show that, using concept profiles, we can detect 43% of the known PPIs, with a specificity of 99%, and 66% of all known PPIs with a specificity of only 95%. In contrast, the direct relations methods and STRING show much lower scores (Table S1).

### Proteins Connected via One Intermediate Protein

The results reported in the previous section indicate that not all proteins with high similarity scores are known to interact according to the combined protein databases. One possible explanation for this is that the proteins are related in another way, *e.g.* they could be involved in the same pathway or be part of the same protein complex, but do not physically interact. To determine whether this occurs, we also tested both concept-based approaches on the detection of known connections via one intermediate protein. For instance, if the protein pairs A-B and B-C are recorded as PPIs in databases, we form the additional protein pair A-C. In total we were able to create 1,028,265 of such pairs to serve as an independent test set. When the pairs are filtered on coverage by all methods the remaining set contains 790,245 pairs. At a specificity level of 99% and 95% the sensitivity level of the different methods was determined for those pairs. The results are given in Table S2 and indicate that the concept profile-based approach is indeed superior in predicting relationships between proteins potentially present in the same complex or pathway.

### Average Prediction Performance per Protein

Most researchers will not be interested in all PPIs, but only in those interactions involving a (set of) protein(s) of interest. Therefore, for each protein we created a top 10, top 100, and top 1,000 best matching proteins according to the concept-based direct relation, the concept profile method, and STRING. In these lists, we calculated the number of PPIs that are either (i) part of the PPI set, or (ii) described in the IntAct database, or else (iii) part of the pairs that are connected through intermediate proteins as described in the previous section. We limited our analyses to the 10,812 proteins that were detected in at least five MEDLINE abstracts (covered by the concept profiles method). The averages of these performance measures in terms of precision and recall are shown in Table S3. For comparison, the average total number of pairs per protein in each set is provided in the third column. For instance, on average each protein is involved in 8.73 interactions according to the PPI set, of which on average 6.34 are found in the top 1,000 of the concept profile method (precision and recall of 0.006 and 0.73 respectively), and only 3.93 and 3.83 in the top 1,000 of the concept-based direct relation method and STRING respectively. The latter two methods show a slightly better performance for the top 10. Thus, it appears that co-occurrence-based methods can detect a smaller number of PPIs with a somewhat higher accuracy, but the concept profile method, by including indirect evidence, can predict more PPIs and is therefore likely to be more valuable for actual knowledge discovery.

### Retrospective Prediction of Currently Known PPIs

Protein annotation databases are struggling to stay up-to-date with the literature, and there is often a substantial time lag between the first publication of a finding, and the time the PPI is entered in a database. It could therefore be postulated that many of the unknown PPIs predicted today are in fact correct, but may not be entered in a database for several years. We have performed a retrospective study to answer the question: how many of the PPIs that would have been predicted by the different methods in 2005 were confirmed in 2007?

Both direct relation and concept profile method-based PPI prediction scores were created using a MEDLINE corpus with publication dates up to February 2005. We ranked the PPIs according to the scores, and set a cut-off value at the 95% and 99% specificity levels based on PPIs present in Swiss-Prot 2005 (this is the only database for which historic versions are available). We subsequently evaluated how many of the 3,295 PPIs that were added to Swiss-Prot between 2005 and 2007 were above these cut-off values in 2005. These are the sensitivity values reported in Table S4. We also calculated the AuC based on Swiss-Prot 2007 alone.

The prediction performance is much better for concept profiles (52% versus 38% for a specificity level of 95%). This indicates that the majority of currently known PPIs were not yet explicitly described in MEDLINE at our testing point, but would have been predicted at a specificity rate of 95%. We postulate that this finding is indicative for the assumption that based on the full current literature a meaningful percentage of the ‘unknowns’ that pass the prediction threshold will be actual pairs worth studying in more detail.

### Case Studies

The next logical step was therefore to investigate whether this method can only predict PPIs that are ‘known’ but not explicit in the literature corpus used, or whether it would also be able to effectively predict unknown, but real PPIs. We investigated this in two case studies. We generated predicted interactions for proteins with two proteins that are intensively investigated in our group: (i) Dystrophin (DMD), a structural protein causing Duchenne muscular dystrophy when defective, and (ii) Calpain 3 (CAPN3), a protease when mutated causing Limb-girdle muscular dystrophy (LGMD).

### DMD

We presented the list of predicted interacting proteins with DMD ordered by descending association scores, to two experts for evaluation. At a specificity of 99%, there are 196 proteins predicted to interact with DMD. This list was too long to manually evaluate and we therefore restricted the human curation analysis to the 99.8% specificity level (top 42 proteins, Table S5). The full list is presented as Table 7 in Supporting Information File S1. The 42 proteins include 7 of the 19 dystrophin-interacting proteins that are known from curated databases (sensitivity of 37% at this very high specificity level). The remaining established interaction partners generally rank high in the list of literature-predicted targets (13/19 in the top 196, p-value from Kolmogorov-Smirnov test for comparison with overall ranking: 3.4 · 10^−10^). There are three proteins in the predicted set with at least indirect evidence in the literature for a physical interaction with DMD (CAV3, SPTB, ACTN2). One protein (SLMAP) may well interact given its distribution and localization but this needs experimental testing. Ten proteins in the list are found in the same protein complex as DMD but do not interact directly as far as known. Four proteins in the list were found wrongly associated with DMD due to homonym problems during literature indexing.

The remaining 17 proteins in the list are associated with DMD for other reasons (e.g. also involved in muscular dystrophy, or structural or functional homology) but are not likely to physically interact. If we only allow direct physical interaction pairs as true positives (11 proteins) the estimated precision is 26%. If predictions of protein pairs in a complex also are counted as true positives (21 proteins in total), the estimated precision would be 50%. Since also conceptually-related proteins that do not physically interact may be of interest to the biologist, the overall precision of our prediction method may be as high as 90%.

### CAPN3

For CAPN3, an evaluation of the precision is more difficult since there is, compared to an intensively studied protein such as DMD, not enough established knowledge about its regulatory partners and substrates. Table S6 summarizes the currently known interaction partners for CAPN3: 13 interactions have been described in the literature (not necessarily in the abstracts that were used for our predictions, see column ‘direct relation’) and of those, six interactions have been entered in PPI databases. These known interaction partners generally rank high in the list of literature-predicted targets (Table S6, p-value from Kolmogorov-Smirnov test: 5.7 · 10^−5^). Interestingly, the concept profiling method correctly predicted the interaction between myosin light chain 1 (MYL1) and CAPN3 on the basis of conceptual overlap in MEDLINE abstracts (specificity >99%), although this interaction was only described in a full text paper [23] and not in any MEDLINE abstract used to generate the concept profiles.

Apart from literature based rediscovery of known interactions, we also set out to actually find new interaction partners for CAPN3. We selected predicted interaction partners that have not been entered in PPI databases so far and that do not have a direct co-occurrence in MEDLINE. The top ranked conceptual match is with Sarcoglycan-epsilon (SGCE), which is the smooth muscle counterpart of SGCA. Like for CAPN3, mutations in SGCA cause LGMD, but as far as we know, the protein is not expressed in skeletal muscle.

The second highest ranking protein was deemed to be an interesting candidate by the experts: Parvalbumin B (PARVB). The concept profiling method yielded a high association score because both proteins are described to have a physical interaction with dysferlin (DYSF) [24], [25], and with α-actinin (ACTN2) [26], [27], and they are both located at the Z-disc [28], [29]. For this predicted protein pair, we experimentally demonstrated a physical interaction, using three different set-ups.

First, it was shown that immobilized GST-fused PARVB could pull down recombinant T7-CAPN3 from bacterial lysates. Second, immobilized GST-PARVB could pull down endogenous CAPN3 from IM2 mouse myoblasts, and vice versa (Figure S2).

CAPN3 is hypothesized to act as a cytoskeleton remodeler and has been shown to interact with other focal adhesion proteins like Talin and Vinexin [30] (see Table S6). Ectopic CAPN3 over-expression results in cell rounding and cleavage and loss of co-expressed Talin and Vinexin [30]. This suggests that CAPN3 is a modulator of focal adhesions. Like CAPN3, PARVB is predominantly expressed in skeletal muscle, where it plays a role in cell spreading and localizes to focal adhesions [26] (for a review, see [31]). The predicted interaction is coherent with this hypothesis, and substantiates the evidence for a role for CAPN3 outside the sarcomere.

This showcase is just one example of a correct and meaningful PPI prediction using concept profiles. This exemplary case study can not be seen proof that many of the other high ranking predictions will also be true physical and biologically relevant interactions. However none of the other consulted applications (STRING, iHOP) predicted this pair of interacting proteins. As the predictions using concept profiling are based on conceptual relatedness rather than an explicit co-occurrence in MEDLINE, this case study is indicative of the power of concept profiles to discover new, implicitly related pairs of interacting proteins. The statistics presented in this paper support the conclusion that predicted PPIs using our method, especially the subset that remains after expert analysis of the top ranking list are likely to be very significantly enriched for proteins that are worthwhile studying in wet lab experiments.

## Discussion

Scientists in general and scientific annotators in particular derive their knowledge on PPIs not directly discovered by their own experiments from the literature. However, as we show here, only 32% of the known PPIs covered by curated PPI databases can be found in MEDLINE abstracts (Table S1), the resource that is most commonly used for concept searches in the biomedical domain. This is despite the use of a sophisticated synonym expansion and homonym disambiguation systems. It is likely that many of these interactions are only mentioned in the full text of articles, or that the interactions have never been explicitly described in literature but were directly submitted to a database. In either case, the applicability of the most commonly used approach for PPI detection - the direct relation method in publicly available literature - appears to be severely limited.

The specificity and sensitivity levels achieved by our novel prediction method appear to be very promising. However, when we predict interaction partners for a specific protein, the estimated precision levels (*i.e*. how many of the predicted proteins are true interaction partners) are still seemingly quite moderate. A first consideration is that we are intrinsically unable to determine an accurate ‘true false positive rate’ for the predicted PPIs, due to the fact that many PPIs have simply not been discovered and described yet. This unavoidable complication most certainly will lead to an underestimation of precision levels. The case study of CAPN3 and PARVB signifies this point; initially this pair would have been classified as a ‘false positive’.

For a realistic estimation of the precision of our prediction method, effectively each predicted protein pair should be validated in a wet lab experiment, which is out of the realistic scope of this study. For this reason we developed Nermal. (http://biosemantics.org/nermal). In Nermal, researchers can enter the UniProt identifier of a protein of interest, and the tool will return a ranked list of proteins that are most likely to interact with the query protein, in combination with information on whether the PPI has already been described explicitly in MEDLINE and/or in one of the protein databases.

A second complicating factor is the size of the ‘negative’ set (>50 million) compared to the ‘positive’ set (44,920). This aspect is illustrated by the average prediction performance for each protein in Table S3 and by the case study with DMD in Table S5, where the top 42 proteins yielded a precision of only 26%, whilst the specificity was 99.8%. We are currently working on a further improvement of the precision by including data sources other than the literature in the PPI prediction algorithms. A final consideration is that our predictions are yielding more conceptual connections than physically interacting proteins only. Conceptual overlap obviously can indicate a variety of other types of relations between proteins. For instance, we demonstrate that many proteins with high concept profile similarity do not interact directly, but are connected through intermediary proteins and are potentially part of the same complex or pathway. Therefore, the precision is to a certain extent dependent on the definition of a useful prediction. When other relationships than direct physical interactions are also deemed of interest, the precision of our method can become as high as 90%. The practical use of concept profiles will be in knowledge discovery in general, which is much broader than discovery of PPIs alone. In fact the hypothetical connection between any given pair of concepts can be calculated using our method.

To allow researchers to incorporate conceptual overlap data into their own analyses, we have made the concept profile similarity scores publicly available in two forms; first, a table containing similarity scores between all human proteins can be downloaded from our website; second, the previous mentioned web tool dubbed Nermal.

We conclude that concept profile similarity is a significantly better literature based predictor of PPIs than co-occurrence based methods. These improved predictions can be used to increase the biological interpretation and accuracy of interaction maps generated by high-throughput experiments, or can be used to prioritize proteins for further testing. In further studies, we will evaluate whether the use of concept profiles can also be applied in the prediction of other types of relations, for instance between drugs and diseases, and between genes and diseases.

## Methods

### Direct Relation Detection

Direct relations are typically extracted from literature based on co-occurrence [32]; if two proteins are mentioned in the same sentence or document more often than can be expected by chance, they are presumably related. We evaluated two alternatives for the detection of protein occurrences: a word-based approach and a concept-based approach. The word-based approach consists of combining the names of two proteins in an ‘AND’ query in the PubMed search engine. For the concept-based approach we have used the concept-recognition software Peregrine [33], [34], which includes synonyms and spelling variations [35] of concepts and uses simple heuristics to resolve homonyms. For this, Peregrine uses a protein ontology that was constructed by combining several gene and protein databases [36]. Even though a previous study has shown that Peregrine achieves state-of-the-art performance (75% precision and 76% recall on the BioCreactive II gene normalization testset [33], [34]), the concept recognition process is still error prone.

We used the likelihood ratio [19] to indicate the strength of the relation between two proteins. This ratio increases with the likelihood of there being a dependency between the occurrence of two proteins. Two hypotheses are used: (i) the occurrence of one protein is statistically dependent on the occurrence of the other protein; (ii) the occurrences are statistically independent. For each hypothesis a likelihood is calculated based on the observed data using the binomial distribution. The ratio of these likelihoods tells us how much more likely one hypothesis is over the other, or, in other words, how sure we are that there is a dependency. The following equations give the likelihood ratio *λ* of concepts *i* and *j*.
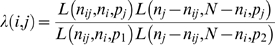
where N is the total number of documents in the corpus, *n_i_*, *n_j_*, and *n_ij_* are the number of documents containing *i, j*, and both *i* and *j*, respectively. 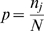, the probability *j* occurs in an abstract irrespective of *i*, 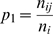, the probability *j* occurs in an abstract containing *i*, 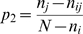, the probability *j* occurs in a document not containing *i*, and 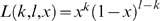, the likelihood function according to the binomial distribution.

### Concept Profile-Based Relation Detection

To calculate the similarity of the contexts in which proteins appear in literature, we summarize the context of each protein in a concept profile. This profile contains all concepts that have a direct relation with a protein as found using the direct relation method described above. We evaluated two possible ways of applying this method: (i) using co-occurrences within a sentence, and (ii) using co-occurrences within an abstract. As shown in supplement S6 (Supporting Information File S1), co-occurrence within an abstract yields a slightly higher AuC on predicting PPIs. We therefore used the abstract-based method in our study. The concepts in a profile include, in addition to proteins, all other concepts described in the Unified Medical Language System (UMLS) [37], such as diseases, symptoms, tissues, biological processes and many other types of concepts. We used the uncertainty coefficient [19] to calculate the weights of the concepts in the profiles. The uncertainty coefficient for the stochastic variables X and Y is given by
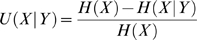
with *H(X)* is the entropy for *X* and *H(X|*Y*)* is the entropy for *X* given Y. X and Y can be any concept known in the ontology, e.g. drugs, proteins, diseases, disorders, chemicals, etc. The uncertainty coefficient is an information-theoretical measure that takes the a priori probability of direct relations into account. It gives extra weight to those concepts that are very specific for the set of documents belonging to the protein for which the concept profile is constructed. For a detailed description of concept profiles we refer to Jelier *et al*. [19].

The similarity score between two concept profiles A and B is taken as the inner product of the concept profile vectors, following Jelier *et al*. [38].
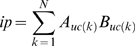
with *uc(k)* the *k^th^* uncertainty coefficient in the profile and N the total number of concepts the two profiles have in common. The inner product increases with increasing overlap in concept profiles. If two proteins co-occur, the inner product of their concept profiles is in general high. This is shown in supplement S4 (Supporting Information File S1).

### MEDLINE Corpus

We extracted the title and abstract of subsections of MEDLINE. The corpus used in our main study has a time span from 1980 up to July 2007 and contains 12,098,042 citations. The corpus used for the retrospective study has a time span from 1980 up to February 2005 and contains 10,363,027 citations. This is an increase in time of 9.8% whereas the increase in published articles over the last two years is 17%.

### Generation of the PPI and NIPP Sets

There are many protein databases that describe PPIs. Not all of these use protein identifiers that could be linked to our protein ontology and the databases also show a high degree of overlap (see supplement S2 in Supporting Information File S1). In our analysis we use BioGRID [39], DIP [40], HPRD [41], IntAct [42], MINT [43], Reactome [44], and Swiss-Prot [45] and only consider human proteins. Except for IntAct, all these databases are curated, meaning that they only contain PPIs that were judged to be correct according to strict criteria. IntAct, on the other hand, also contains unchecked results from high-throughput experiments which could contain many false positives. For a comparison of the prediction performance of our method on the individual databases we refer to supplement S3 (Supporting Information File S1). The release dates and dates of download can be found in supplement S1 (Supporting Information File S1).

For the construction of our set of known PPIs, we only rely on the curated databases; if a PPI was mentioned in one of these databases, we assumed it to be a true PPI. The resulting positive set contains 61,807 PPIs. After removing pairs that are not covered by all four prediction methods, 44,920 PPIs remain. Unfortunately, there is no database of proteins that are known not to interact. We can therefore only create a set of proteins which are less likely to interact. For our NIPP set we took all pairs of human proteins that are not in the PPI set, and are not in the high-throughput part of the IntAct database. For computational reasons the calculation of the specificity and AuC was done on a random sample of 44,920 pairs of this set, setting both the positive and negative set size equal. Two randomly selected proteins form a pair and are checked if (i) they are not in the positive PPI set, (ii) not the same protein, e.g. proteins that interact with themselves are not taken into account, (iii) the protein pair is not already in the NIPP set, e.g. protein pairs can only occur once in a set. The random sample is actually quite small compared to the total NIPP set, however the ROC curve analysis is set size independent if the sample size is sufficiently large.

One last remark is that the positive set is incomplete. Therefore the creation of the NIPP set will introduce false negatives (PPIs that should have been in the positive set and recorded in a curated database). However the bias introduced by false negatives is negligible since the ratio of expected PPIs in human compared to the total set of formable protein pairs (∼60 million) is very small [22].

### STRING Database

A copy of the STRING database, version 7.1, was downloaded from the STRING website. STRING is a pre-calculated database in PostgreSQL format. Only the text mining score table was used in our analysis.

### Sensitivity, Specificity, Precision

In information retrieval terms like the sensitivity, specificity and precision are frequently used. The definitions are:
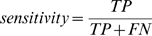


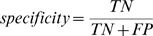


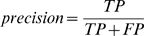
where TP are the number of true positives, FN number of false negatives, FP number of false positives, and TN number of true negatives. A perfect predictor has a specificity and sensitivity of 1.

When both set sizes are equal (#NIPP = #PPI) the precision equals the sensitivity. The specificity is sometimes confused with the precision. The distinction is critical when the classes are different sizes. A test with very high specificity can have a very low precision if there are far more true negatives than true positives, and vice versa.

### Online Web Tool Nermal

Nermal is a web tool that prioritizes proteins that are most likely to be related with the protein you study. Given a query protein, the similarity scores are calculated between this protein and all other proteins in the ontology. The proteins are ranked on the similarity scores and presented in a table. Each row shows the similarity score between the two proteins, the databases in which the protein pair is known, and the sensitivity and (1-specificity) for that similarity score. These two rates should be interpreted as follows: given a similarity score between two proteins, (1-specificity) is the probability that a protein pair passing that score is a false positive. The sensitivity is the probability that you will miss a true PPI at that same score. Nermal can be found on http://biosemantics.org/nermal/. The full set of all protein pair match scores for human proteins can be downloaded at this link as well as the PPI and NIPP set used in the study.

### DNA Cloning

PARVB was amplified from proliferating IM2 myoblast cDNA with the following UTR primers: fw cgcactcgcttatgtcctc, rv ctccacatccttgtacttggtg. The ORF was amplified with a nested PCR introducing restriction sites for cloning into pET28aGST (modified pET28a vector with GST tag instead of T7 [46]). Primers were: fw aatatggatcctcctccgcgccaccacggt, rv atattctcgagctccacatccttgtacttgg. CAPN3 was similarly amplified with primers fw atgccaactgttattagtc, and rv ctaggcatacatggtaagc, and cloned into pET28aGST using fw tattacggatccatgccaactgttattagtc, and rv gtaatactcgagctaggcatacatggtaagc. The exon 6 deletion that does not autolyse was used for this experiment.

CAPN3c129s in pET28c was described previously [47]. All DNA constructs were verified by direct sequencing (LGTC, Leiden, The Netherlands), and subsequently transformed into BL21 (DE3)-RIL *E. coli* cells (Stratagene) for protein production.

### Protein Production and Preparation of Lysates

BL21 cells transformed with pET28aGST, pET28aGST-PARVB, pET28aGST-CAPN3 or pET28cCAPN3c129s were grown to log phase and stimulated with 1 mM IPTG (Fermentas), and left to grow for 3 h at 37°C. Next cells were spun down at 3,000 g and 4°C for 15 min. Pellets were dissolved in lysis buffer A (50 mM Tris-HCl pH 7.4, 1 mM EDTA, 1.5 mg/ml lysozyme, 0.15 M NaCl, 1% Triton, Benozonase, 2x protease inhibitor cocktail tablet (Roche Molecular Biochemicals, Basel, Switzerland)), and sonicated on ice. Lysate was cleared by centrifugation at 13,000 g, and 4°C for 30 min.

IM2 cells were grown at 33°C and 10% CO2 in DMEM 60196 (GIBCO-BRL, Grand-Island, NY) supplemented with 20% FCS, INFγ, glucose, pen/strep, glutamine and chick embryo extract. 15 cm plates (2x) were grown 75% confluent, washed 1x with PBS (37°C) and lysed on ice with 1 ml lysis buffer B (50 mM Tris-HCl pH 7.5, 150 mM NaCl, 0.2% Triton X-100, 2x protease inhibitor cocktail tablet). Lysate was spun down at 13,000 g and 4°C for 30 min.

### Pull-Down

GST sepharose beads (4B, Amersham, Uppsala, Sweden) were washed with PBS (2x) and pre-equilibrated with lysis buffer (2x), and added to the cleared GST fusion lysates. Lysates were incubated at 4°C and tumbling for 2 h. Next the lysates were spun down at 500 g, 4°C for 5 min, and washed 3x with lysis buffer A. Separately, IM2 lysates were treated with washed and pre-equilibrated GST sepharose beads (buffer B). An aliquot of the GST fusion proteins was loaded on SDS-PAGE gel and Coomassie stained to confirm equal loading.

IM2 lysate, or T7-CAPN3c129s lysate, was added to the bait, and incubated O/N at 4°C and tumbling. GST sepharose beads were spun down and the sup was stored as non-bound fraction. The beads were washed 5x with ice cold lysisbuffer (A or B, 3x short, 2x five minutes tumbling). All remaining sup was removed with an insulin syringe and proteins were eluted with 2x Laemmli sample buffer and boiled 5 min. An aliquot of the non-bound fraction was similarly prepared.

### Western Blot

Samples were loaded onto SDS-PAGE gels, separated and blotted to PVDF membrane. Blots were blocked in 4% skimmed milk PBS (Marvel) and incubated with primary antibody O/N at 4°C. Next morning blots were washed with 0.05% Tween in PBS, and incubated with secondary antibody for 1 h. Blots were washed again and scanned with an Odyssey scanner (Licor) or incubated with ECL plus (Amersham) and exposed to a Kodak XAR film. The following antibodies were used for Western detection: GaGST (1;10,000 Stratagene) MaCAPN3 (1;100, 12A2 Novocasta, Newcastle, UK), GaPARVB (1;200 Santa Cruz), GaMouseIRDye680 (1;5,000 Westburg, Leusden, NL), DaGIRDye800 (1;5,000 Westburg), RaMouseHRP (1;2,000 Dako Cytomation, Glostrup, Denmark), DaGoatHRP (1;10,000 Promega).

## Supporting Information

Figure S1 Histogram of the distributions of similarity scores of the concept profile-based method for the PPI and NIPP sets. A log transformation is applied to the similarity scores for better visualization.
(1.35 MB TIF)

Figure S2 CAPN3 and PARVB can directly interact. A: Immobilized GST-fused PARVB can pull down recombinant CAPN3 from a bacterial T7-tagged CAPN3 lysate (Lane 2 vs 1), where unfused GST cannot (Lane 4 vs 3). As CAPN3 is an unstable protein that outside skeletal muscle rapidly autolyses we used the active site mutant C129S48. All fractions were resolved on SDS-PAGE gel and analyzed by immunoblotting with anti-CAPN3. The lanes represent: GST-PARVB non-bound fraction (1), GST-PARVB bound fraction (2), GST non-bound fraction (3), GST bound fraction (4). B: Equal loading was confirmed with anti-GST (Lane 1 GST-PARVB, Lane 2 GST). C: GST-fused PARVB can pull down endogenous full-length CAPN3 from an IM2 lysate (Lane 1 vs 2), contrary to unfused GST (Lane 3 vs 4). Lane 1 GST-PARVB bound fraction, Lane 2 non-bound fraction, Lane 3 GST bound fraction, Lane 4 non bound fraction. D: Likewise, GST-CAPN3 can pull down endogenous PARVB (Lane 1), contrary to GST (Lane 2). Both PARVB translation products bind. Here we used the Δ6 variant of Capn3 that does not autolyse yet retains function30, 49, and is expressed in the proliferating IM2 myoblasts. The arrows indicate the detected proteins and in all panels a molecular marker is depicted on the left.
(1.67 MB TIF)

Table S1 Performance of different PPI prediction approaches on detecting known PPIs in MEDLINE. CDR stands for Concept-based Direct Relation method.
(0.03 MB DOC)

Table S2 Performance on predicting proteins that are connected via an intermediate protein.
(0.03 MB DOC)

Table S3 Analysis of the top 10, 100, and 1,000 returned by the Concept Profile (CP) method, the Concept-based Direct Relation (CDR) method, and by STRING. The analysis shows the precision and recall of protein pairs that are in the PPI set, of additional pairs that are found in IntAct, and of additional pairs that are in the set of protein pairs that are connected via an intermediate protein. In the field of information retrieval the term recall is more often used instead of sensitivity.
(0.04 MB DOC)

Table S4 Results of the retrospective prediction of PPIs added to Swiss-Prot between 2005 and 2007. PPIs are ranked based on MEDLINE up to 2005, and specificity levels are based on Swiss-Prot 2005.The sensitivity is determined on Swiss-Prot 2007.
(0.03 MB DOC)

Table S5 Top 42 ranked proteins with DMD. In total 10,812 proteins were matched against DMD. 7 proteins as known to interact with DMD. Only 4 proteins are real false positives due to homonyms problem resulting in a precision over 0.9.
(0.09 MB DOC)

Table S6 List of proteins known to interact with Calpain-3. In total 10,812 proteins known to have a concept profile are matched against Calpain-3.
(0.05 MB DOC)

Supporting Information File S1 Supplementary data.
(0.59 MB DOC)
