# Supplementary figures and images for: Novel Protein-Protein Interactions Inferred from Literature Context
Source: PLoS One. 2009 Nov 18;4(11):e7894. doi: 10.1371/journal.pone.0007894 (PMC2774517; doi:10.1371/journal.pone.0007894)

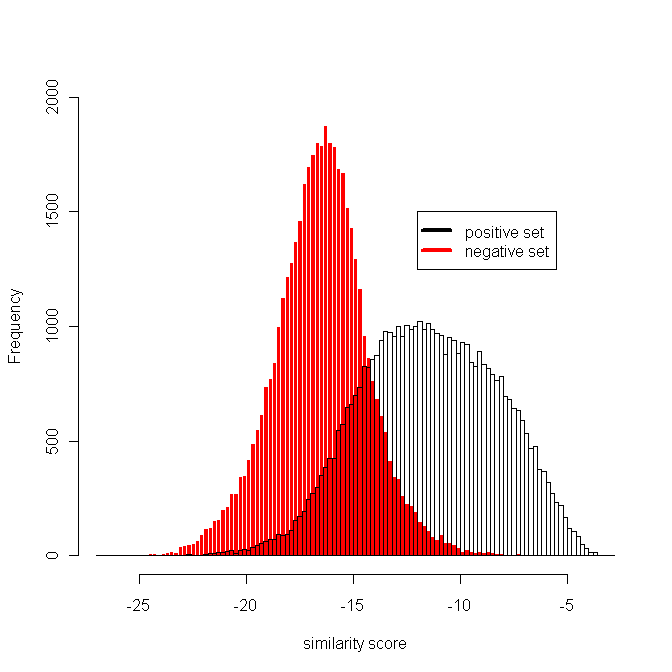

Supplement: Figure S1 — Histogram of the distributions of similarity scores of the concept profile-based method for the PPI and NIPP sets. A log transformation is applied to the similarity scores for better visualization. (1.35 MB TIF) [file pone.0007894.s001.tif]

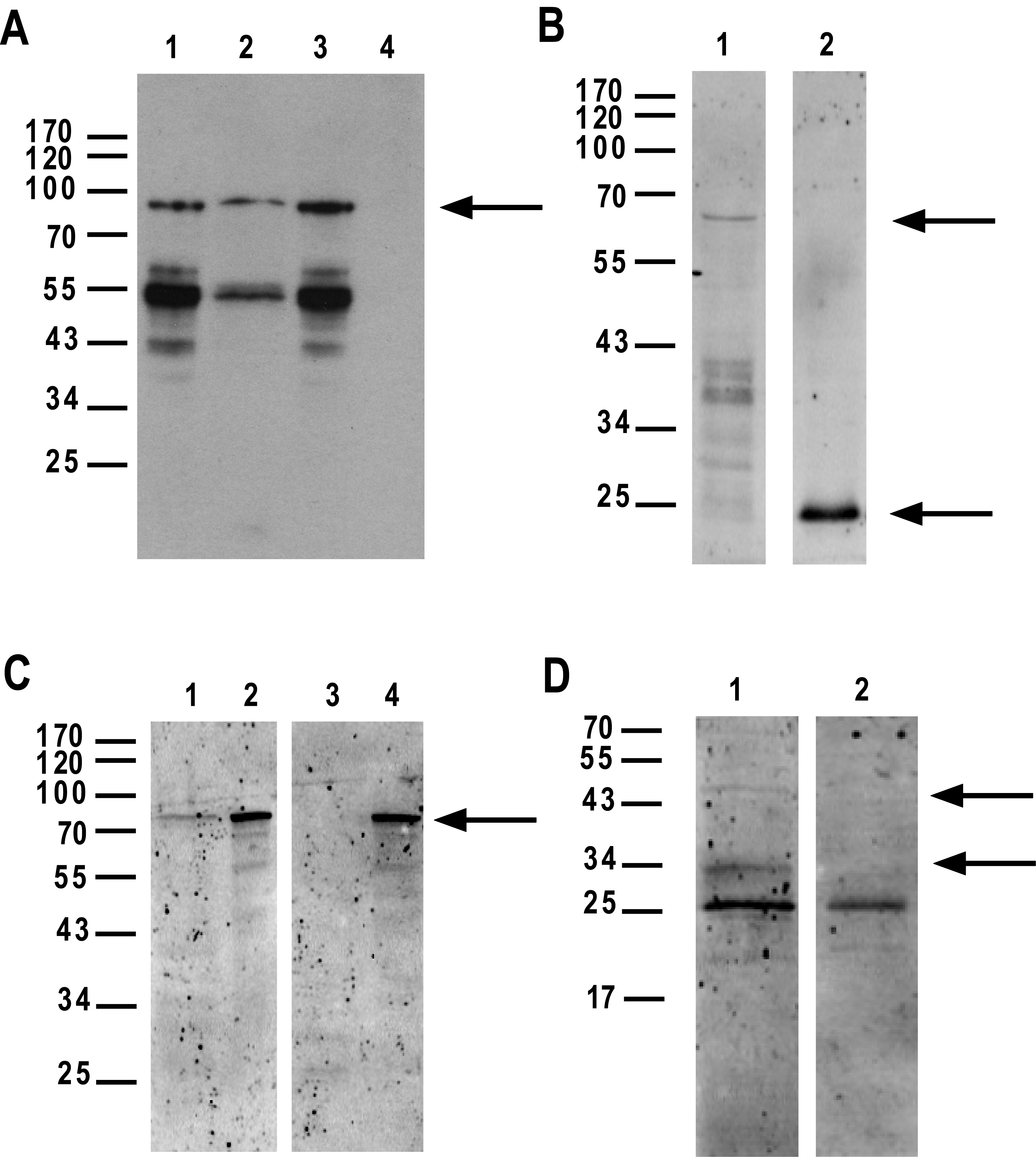

Supplement: Figure S2 — CAPN3 and PARVB can directly interact. A: Immobilized GST-fused PARVB can pull down recombinant CAPN3 from a bacterial T7-tagged CAPN3 lysate (Lane 2 vs 1), where unfused GST cannot (Lane 4 vs 3). As CAPN3 is an unstable protein that outside skeletal muscle rapidly autolyses we used the active site mutant C129S48. All fractions were resolved on SDS-PAGE gel and analyzed by immunoblotting with anti-CAPN3. The lanes represent: GST-PARVB non-bound fraction (1), GST-PARVB bound fraction (2), GST non-bound fraction (3), GST bound fraction (4). B: Equal loading was confirmed with anti-GST (Lane 1 GST-PARVB, Lane 2 GST). C: GST-fused PARVB can pull down endogenous full-length CAPN3 from an IM2 lysate (Lane 1 vs 2), contrary to unfused GST (Lane 3 vs 4). Lane 1 GST-PARVB bound fraction, Lane 2 non-bound fraction, Lane 3 GST bound fraction, Lane 4 non bound fraction. D: Likewise, GST-CAPN3 can pull down endogenous PARVB (Lane 1), contrary to GST (Lane 2). Both PARVB translation products bind. Here we used the Δ6 variant of Capn3 that does not autolyse yet retains function30, 49, and is expressed in the proliferating IM2 myoblasts. The arrows indicate the detected proteins and in all panels a molecular marker is depicted on the left. (1.67 MB TIF) [file pone.0007894.s002.tif]
