# Supplementary material for: Novel Protein-Protein Interactions Inferred from Literature Context
Source: PLoS One. 2009 Nov 18;4(11):e7894. doi: 10.1371/journal.pone.0007894 (PMC2774517; doi:10.1371/journal.pone.0007894)
Supplement: Table S1 — Performance of different PPI prediction approaches on detecting known PPIs in MEDLINE. CDR stands for Concept-based Direct Relation method. (0.03 MB DOC) [file pone.0007894.s003.doc]

|  | Word-based | CDR | Concept profiles | STRING |
| --- | --- | --- | --- | --- |
| Sensitivity at spec = 99% | 28% | 37% | 43% | 39% |
| Sensitivity at spec = 95% | 33% | 41% | 66% | 41% |
| Area under Curve | 0.62 | 0.69 | 0.90 | 0.69 |
