# Supplementary material for: Novel Protein-Protein Interactions Inferred from Literature Context
Source: PLoS One. 2009 Nov 18;4(11):e7894. doi: 10.1371/journal.pone.0007894 (PMC2774517; doi:10.1371/journal.pone.0007894)
Supplement: Table S2 — Performance on predicting proteins that are connected via an intermediate protein. (0.03 MB DOC) [file pone.0007894.s004.doc]

|  | Concept-based | CDR | STRING |
| --- | --- | --- | --- |
| Sensitivity at spec = 99% | 8% | 9% | 8% |
| Sensitivity at spec = 95% | 13% | 29% | 12% |
| Area under Curve | 0.54 | 0.78 | 0.53 |
