# Supplementary material for: Novel Protein-Protein Interactions Inferred from Literature Context
Source: PLoS One. 2009 Nov 18;4(11):e7894. doi: 10.1371/journal.pone.0007894 (PMC2774517; doi:10.1371/journal.pone.0007894)
Supplement: Table S3 — Analysis of the top 10, 100, and 1,000 returned by the Concept Profile (CP) method, the Concept-based Direct Relation (CDR) method, and by STRING. The analysis shows the precision and recall of protein pairs that are in the PPI set, of additional pairs that are found in IntAct, and of additional pairs that are in the set of protein pairs that are connected via an intermediate protein. In the field of information retrieval the term recall is more often used instead of sensitivity. (0.04 MB DOC) [file pone.0007894.s005.doc]

|  |  |  | Top 10 | | Top 100 | | Top 1,000 | |
| --- | --- | --- | --- | --- | --- | --- | --- | --- |
|  | Method | Total | Precision | Recall | Precision | Recall | Precision | Recall |
| PPI | CP | 8.73 | 0.096 | 0.110 | 0.033 | 0.37 | 0.006 | 0.73 |
|  | CDR | 8.73 | 0.108 | 0.124 | 0.026 | 0.30 | 0.004 | 0.45 |
|  | STRING | 8.73 | 0.112 | 0.128 | 0.026 | 0.30 | 0.004 | 0.44 |
| IntAct | CP | 1.61 | 0.009 | 0.056 | 0.002 | 0.12 | 0.000 | 0.29 |
|  | CDR | 1.61 | 0.009 | 0.056 | 0.002 | 0.11 | 0.000 | 0.24 |
|  | STRING | 1.61 | 0.008 | 0.050 | 0.002 | 0.11 | 0.000 | 0.24 |
| Indirectly connected | CP | 190.21 | 0.105 | 0.006 | 0.080 | 0.042 | 0.048 | 0.25 |
|  | CDR | 190.21 | 0.137 | 0.007 | 0.068 | 0.036 | 0.027 | 0.14 |
|  | STRING | 190.21 | 0.100 | 0.005 | 0.062 | 0.033 | 0.026 | 0.14 |
