# Supplementary material for: Novel Protein-Protein Interactions Inferred from Literature Context
Source: PLoS One. 2009 Nov 18;4(11):e7894. doi: 10.1371/journal.pone.0007894 (PMC2774517; doi:10.1371/journal.pone.0007894)
Supplement: Table S4 — Results of the retrospective prediction of PPIs added to Swiss-Prot between 2005 and 2007. PPIs are ranked based on MEDLINE up to 2005, and specificity levels are based on Swiss-Prot 2005.The sensitivity is determined on Swiss-Prot 2007. (0.03 MB DOC) [file pone.0007894.s006.doc]

|  | Concept-based | Concept profiles |
| --- | --- | --- |
| Sensitivity at spec = 99% | 27% | 33% |
| Sensitivity at spec = 95% | 38% | 52% |
| Area under Curve | 0.70 | 0.84 |
