# Supplementary material for: Novel Protein-Protein Interactions Inferred from Literature Context
Source: PLoS One. 2009 Nov 18;4(11):e7894. doi: 10.1371/journal.pone.0007894 (PMC2774517; doi:10.1371/journal.pone.0007894)
Supplement: Table S5 — Top 42 ranked proteins with DMD. In total 10,812 proteins were matched against DMD. 7 proteins as known to interact with DMD. Only 4 proteins are real false positives due to homonyms problem resulting in a precision over 0.9. (0.09 MB DOC) [file pone.0007894.s007.doc]

| **Rank** | **Protein symbol** | **Swiss-Prot id** | **Log similarity score** | **Direct relations** | **In PPI set** | **False positives (homonym)** |
| --- | --- | --- | --- | --- | --- | --- |
| 1 | **UTRN** | P46939 | -5.14 | 214 | x |  |
| 2 | SGCA | Q16586 | -6.13 | 119 |  |  |
| 3 | **DAG1** | Q14118 | -6.22 | 139 | x |  |
| 4 | SGCB | Q16585 | -6.60 | 54 |  |  |
| 5 | SGCD | Q53XA5 | -6.95 | 46 |  |  |
| 6 | FCMD | O75072 | -7.05 | 29 |  |  |
| 7 | DYSF | O75923 | -7.19 | 43 |  |  |
| 8 | **DTNA** | Q9BS59 | -7.31 | 17 | x |  |
| 9 | DRP2 | Q13474 | -7.34 | 9 |  |  |
| 10 | SSPN | Q0JV68 | -7.45 | 17 |  |  |
| 11 | LAMA2 | P24043 | -7.46 | 25 |  |  |
| 12 | GK1 | P32189 | -7.56 | 33 |  | x |
| 13 | CAPN3 | P20807 | -7.93 | 28 |  |  |
| 14 | CAV3 | P56539 | -7.95 | 24 |  |  |
| 15 | **SNTA1** | Q13424 | -7.97 | 8 | x |  |
| 16 | EIF3S12 | Q9UBQ5 | -8.05 | 91 |  | x |
| 17 | BEST1 | O76090 | -8.13 | 26 |  | x |
| 18 | SPTB | P11277 | -8.15 | 15 |  |  |
| 19 | FKRP | Q9H9S5 | -8.16 | 4 |  |  |
| 20 | MEB | 6988 | -8.17 | 7 |  |  |
| 21 | SLMAP | Q14BN4 | -8.20 | 4 |  |  |
| 22 | **SNTB1** | Q13884 | -8.20 | 6 | x |  |
| 23 | NEB | P20929 | -8.33 | 16 |  |  |
| 24 | SGCE | O43556 | -8.35 | 10 |  |  |
| 25 | SGCG | Q13326 | -8.46 | 305 |  |  |
| 26 | ACTN2 | P35609 | -8.49 | 11 |  |  |
| 27 | POMT1 | Q5JT03 | -8.50 | 3 |  |  |
| 28 | LOC130074 | Q6NZ40 | -8.50 | 16 |  | x |
| 29 | CMD1K | 14541 | -8.50 | 27 |  |  |
| 30 | FER1L3 | Q9NZM1 | -8.51 | 3 |  |  |
| 31 | NOS1 | P29475 | -8.53 | 42 |  |  |
| 32 | IKBKAP | O95163 | -8.63 | 10 |  |  |
| 33 | MACF1 | Q5T3B3 | -8.66 | 9 |  |  |
| 34 | AQP4 | P55087 | -8.67 | 13 |  |  |
| 35 | CKM | P06732 | -8.70 | 11 |  |  |
| 36 | FSHMD1A | 3966 | -8.74 | 8 |  |  |
| 37 | TCAP | O15273 | -8.75 | 7 |  |  |
| 38 | **DTNB** | O60941 | -8.76 | 9 | x |  |
| 39 | LOC619409 | 619409 | -8.82 | 5 |  |  |
| 40 | VCL | P18206 | -8.87 | 36 |  |  |
| 41 | LGMD1A | 6574 | -8.88 | 3 |  |  |
| 42 | **SNTG1** | Q9NSN8 | -8.90 | 5 | x |  |
