# Supplementary material for: Novel Protein-Protein Interactions Inferred from Literature Context
Source: PLoS One. 2009 Nov 18;4(11):e7894. doi: 10.1371/journal.pone.0007894 (PMC2774517; doi:10.1371/journal.pone.0007894)
Supplement: Table S6 — List of proteins known to interact with Calpain-3. In total 10,812 proteins known to have a concept profile are matched against Calpain-3. (0.05 MB DOC) [file pone.0007894.s008.doc]

| Name | Symbol | In PPI set | In literature  (full text) | Direct relation  (abstract) | Rank in literature-based prediction | Significant at specificity of 95 % |
| --- | --- | --- | --- | --- | --- | --- |
| Dysferlin | DYSF | x | x | x | 2 | x |
| Titin | TTN | x | x | x | 4 | x |
| Filamin C | FLNC | x | x | x | 27 | x |
| Alpha-actinin | ACTN2 |  | x | x | 43 | x |
| Calpastatin | CAST |  | x | x | 55 | x |
| IkappaBalpha | NFKBIA | x | x | x | 126 | x |
| Myosin light chain 1 | MYL1 |  | x |  | 398 | x |
| Alpha-spectrin | SPTAN1 | x | x |  | 426 | x |
| Filamin A | FLNA |  | x |  | 853 |  |
| Ezrin | VIL2 |  | x |  | 2739 |  |
| Vinexin | SORBS3 |  | x |  | 3301 |  |
| Talin | TLN1 |  | x |  | 4725 |  |
| AHNAK | AHNAK |  | x | No (*) | 7371 |  |
| YWHAQ | YWHAQ | x |  |  | 7617 |  |

(*) paper describing this interaction in the abstract appeared in June 2008 and was not in the literature corpus used for the prediction
