# Supplementary material for: Novel Protein-Protein Interactions Inferred from Literature Context
Source: PLoS One. 2009 Nov 18;4(11):e7894. doi: 10.1371/journal.pone.0007894 (PMC2774517; doi:10.1371/journal.pone.0007894)
Supplement: Supporting Information File S1 — Supplementary data. (0.59 MB DOC) [file pone.0007894.s009.doc]

**Supplementary information belonging to the article “Novel protein-protein interactions inferred from literature context”**

**S1 Downloaded protein database and release dates**

In total seven protein databases are used in the study. The UniProt database consists of Swiss-Prot and TrEMBL.

| Protein database | Date of download |
| --- | --- |
| Biogrid | September 28, 2007 |
| DIP | September 20, 2007 |
| HPRD | August 22, 2007* |
| IntAct | January 26, 2008 |
| MINT | September 24, 2007* |
| Reactome | September 20, 2007 |
| UniProt | February 14, 2008* |

* For these databases it is possible to retrieve the original release dates. HPRD was released at January 9, 2007, MINT at June 28, 2007. Swiss-Prot and TrEMBL are combined in the database UniProt and have different release versions. UniProt release 12.0 contains Swiss-Prot release 54.0 and TrEMBL release 37.0. Both are dated from July 24, 2007.

**S2 PPI overlap between the seven databases**

Many of the PPIs appear in several databases. The following table shows the distribution and overlap over the seven protein databases.

|  | Biogrid | DIP | HPRD | IntAct | MINT | Reactome | Swiss-Prot |
| --- | --- | --- | --- | --- | --- | --- | --- |
| Biogrid | **16240** | 205 | 15476 | 3006 | 2637 | 909 | 827 |
| DIP |  | **365** | 278 | 84 | 118 | 66 | 53 |
| HPRD |  |  | **34957** | 8031 | 7046 | 1401 | 1839 |
| IntAct |  |  |  | **17456** | 5754 | 595 | 3839 |
| MINT |  |  |  |  | **10772** | 375 | 650 |
| Reactome |  |  |  |  |  | **29672** | 290 |
| Swiss-Prot |  |  |  |  |  |  | **3841** |

**S3 Performance on individual databases**

The positive set is a combination of six protein databases. The databases vary in size and also the level of curation of each PPI. The following table gives the Area under the ROC (AuC) curve for each database individually. The last row is the AuC for the complete positive set.

| Database | Concept profiles | Log likelihood | String |
| --- | --- | --- | --- |
| Biogrid | 0.95 | 0.82 | 0.82 |
| Dip | 0.99 | 0.96 | 0.94 |
| Hprd | 0.93 | 0.79 | 0.78 |
| Intact | 0.71 | 0.57 | 0.56 |
| Mint | 0.87 | 0.72 | 0.70 |
| Reactome | 0.90 | 0.60 | 0.60 |
| Swiss-Prot | 0.84 | 0.71 | 0.71 |
| Positive set | 0.90 | 0.69 | 0.69 |

**S4 Relationship between direct relation detection and concept profiles**

The coverage in S3 shows that some PPIs have both overlap in concept profiles and a direct relation, while others have only concept profile overlap. The similarity score for proteins that share a direct relation is generally high. This is illustrated in figure 1.

Figure 1. Histogram of the distribution of the similarity scores of: (blue) PPIs with concept profile overlap and no direct relation, and (green) PPIs with both a concept profile overlap and a direct relation.

**S5 ROC curve analysis**

The next figure shows the ROC curves for the concept profile similarity score (green), and the likelihood ratio of the direct relation method (red). For the direct relation method we discern two special cases: (i) each protein individual occurs in Medline but they are never mentioned together, and (ii) one of the proteins does not occur in MedLine at all. In the first case the likelihood score is –infinity, in the second case the likelihood score is 0. These cases are quite frequent resulting in many duplicate values, and no natural ordering of the PPIs. We assume a perfect random ordering, resulting in the straight line at the end of the ROC curve in the figure (red for concept based method and black for the String database).


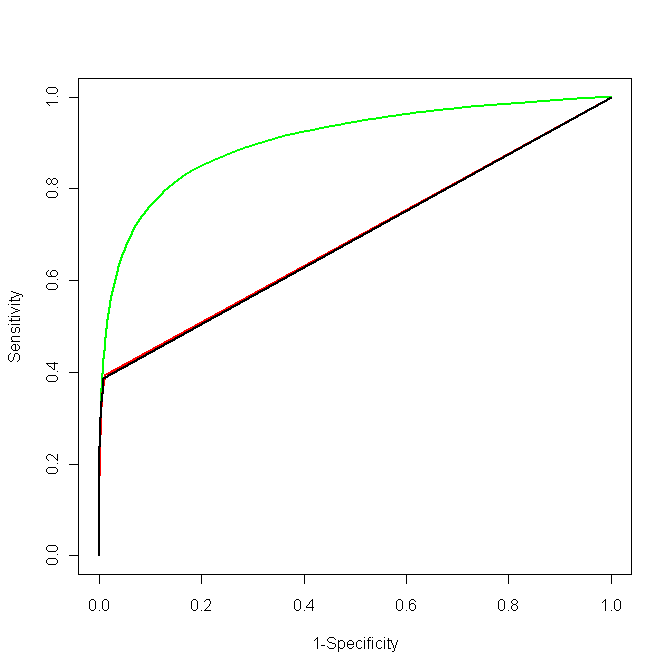


**S6 Relation detection at the abstract and sentence level**

For the construction of concept profiles, we investigated two options: assume two concepts are related when they co-occur (i) in the same sentence, and (ii) in the same abstract. For each option we evaluated the performance on the prediction of PPIs.

|  | Abstract level | Sentence level |
| --- | --- | --- |
| *AuC** | 0.93 | 0.91 |

The difference in results are neglectable. There is a very small decrease in performance using sentence based detection of relations.

* this analysis was done using a MedLine corpus up to April 2007 and using an older ontology.

**S7 Ranked list of proteins predicted to interact with dystrophin (DMD)**

The following table shows the proteins which similarity score with DMD have a specificity higher than 99%.

| **Rank** | **Protein symbol** | **Swiss-Prot id** | **Log similarity score** | **Direct relations** | **FP rate** | **TP rate** | **Biogrid** | **Dip** | **Hprd** | **Intact** | **Mint** | **Reactome** | **Swiss-Prot** |
| --- | --- | --- | --- | --- | --- | --- | --- | --- | --- | --- | --- | --- | --- |
| 1 | UTRN | P46939 | -5.14 | 214 | 0.003 | 0.856 | 0 | 0 | 1 | 0 | 0 | 0 | 0 |
| 2 | SGCA | Q16586 | -6.13 | 119 | 0.013 | 4.047 | 0 | 0 | 0 | 0 | 0 | 0 | 0 |
| 3 | DAG1 | Q14118 | -6.22 | 139 | 0.013 | 4.047 | 0 | 0 | 1 | 0 | 0 | 0 | 0 |
| 4 | SGCB | Q16585 | -6.60 | 54 | 0.022 | 5.853 | 0 | 0 | 0 | 0 | 0 | 0 | 0 |
| 5 | SGCD | Q53XA5 | -6.95 | 46 | 0.032 | 8.168 | 0 | 0 | 0 | 0 | 0 | 0 | 0 |
| 6 | FCMD | O75072 | -7.05 | 29 | 0.034 | 8.620 | 0 | 0 | 0 | 0 | 0 | 0 | 0 |
| 7 | DYSF | O75923 | -7.19 | 43 | 0.039 | 9.650 | 0 | 0 | 0 | 0 | 0 | 0 | 0 |
| 8 | DTNA | Q9BS59 | -7.31 | 17 | 0.048 | 10.576 | 0 | 0 | 1 | 0 | 1 | 0 | 0 |
| 9 | DRP2 | Q13474 | -7.34 | 9 | 0.049 | 10.625 | 0 | 0 | 0 | 0 | 0 | 0 | 0 |
| 10 | SSPN | Q0JV68 | -7.45 | 17 | 0.055 | 11.543 | 0 | 0 | 0 | 0 | 0 | 0 | 0 |
| 11 | LAMA2 | P24043 | -7.46 | 25 | 0.055 | 11.543 | 0 | 0 | 0 | 0 | 0 | 0 | 0 |
| 12 | GK1 | P32189 | -7.56 | 33 | 0.059 | 12.306 | 0 | 0 | 0 | 0 | 0 | 0 | 0 |
| 13 | CAPN3 | P20807 | -7.93 | 28 | 0.080 | 15.060 | 0 | 0 | 0 | 0 | 0 | 0 | 0 |
| 14 | CAV3 | P56539 | -7.95 | 24 | 0.080 | 15.060 | 0 | 0 | 0 | 0 | 0 | 0 | 0 |
| 15 | SNTA1 | Q13424 | -7.97 | 8 | 0.081 | 15.274 | 0 | 0 | 1 | 0 | 0 | 0 | 0 |
| 16 | EIF3S12 | Q9UBQ5 | -8.05 | 91 | 0.091 | 16.020 | 0 | 0 | 0 | 0 | 0 | 0 | 0 |
| 17 | BEST1 | O76090 | -8.13 | 26 | 0.096 | 16.703 | 0 | 0 | 0 | 0 | 0 | 0 | 0 |
| 18 | SPTB | P11277 | -8.15 | 15 | 0.097 | 16.896 | 0 | 0 | 0 | 0 | 0 | 0 | 0 |
| 19 | FKRP | Q9H9S5 | -8.16 | 4 | 0.098 | 17.046 | 0 | 0 | 0 | 0 | 0 | 0 | 0 |
| 20 | MEB | 6988 | -8.17 | 7 | 0.099 | 17.106 | 0 | 0 | 0 | 0 | 0 | 0 | 0 |
| 21 | SLMAP | Q14BN4 | -8.20 | 4 | 0.102 | 17.288 | 0 | 0 | 0 | 0 | 0 | 0 | 0 |
| 22 | SNTB1 | Q13884 | -8.20 | 6 | 0.102 | 17.288 | 0 | 0 | 1 | 1 | 0 | 0 | 1 |
| 23 | NEB | P20929 | -8.33 | 16 | 0.117 | 18.497 | 0 | 0 | 0 | 0 | 0 | 0 | 0 |
| 24 | SGCE | O43556 | -8.35 | 10 | 0.117 | 18.497 | 0 | 0 | 0 | 0 | 0 | 0 | 0 |
| 25 | SGCG | Q13326 | -8.46 | 305 | 0.132 | 19.584 | 0 | 0 | 0 | 0 | 0 | 0 | 0 |
| 26 | ACTN2 | P35609 | -8.49 | 11 | 0.137 | 19.754 | 0 | 0 | 0 | 0 | 0 | 0 | 0 |
| 27 | POMT1 | Q5JT03 | -8.50 | 3 | 0.137 | 19.754 | 0 | 0 | 0 | 0 | 0 | 0 | 0 |
| 28 | LOC130074 | Q6NZ40 | -8.50 | 16 | 0.138 | 19.925 | 0 | 0 | 0 | 0 | 0 | 0 | 0 |
| 29 | CMD1K | 14541 | -8.50 | 27 | 0.138 | 19.925 | 0 | 0 | 0 | 0 | 0 | 0 | 0 |
| 30 | FER1L3 | Q9NZM1 | -8.51 | 3 | 0.138 | 19.925 | 0 | 0 | 0 | 0 | 0 | 0 | 0 |
| 31 | NOS1 | P29475 | -8.53 | 42 | 0.139 | 20.110 | 0 | 0 | 0 | 0 | 0 | 0 | 0 |
| 32 | IKBKAP | O95163 | -8.63 | 10 | 0.152 | 21.011 | 0 | 0 | 0 | 0 | 0 | 0 | 0 |
| 33 | MACF1 | Q5T3B3 | -8.66 | 9 | 0.162 | 21.337 | 0 | 0 | 0 | 0 | 0 | 0 | 0 |
| 34 | AQP4 | P55087 | -8.67 | 13 | 0.162 | 21.337 | 0 | 0 | 0 | 0 | 0 | 0 | 0 |
| 35 | CKM | P06732 | -8.70 | 11 | 0.167 | 21.668 | 0 | 0 | 0 | 0 | 0 | 0 | 0 |
| 36 | FSHMD1A | 3966 | -8.74 | 8 | 0.172 | 21.859 | 0 | 0 | 0 | 0 | 0 | 0 | 0 |
| 37 | TCAP | O15273 | -8.75 | 7 | 0.173 | 22.153 | 0 | 0 | 0 | 0 | 0 | 0 | 0 |
| 38 | DTNB | O60941 | -8.76 | 9 | 0.173 | 22.153 | 0 | 0 | 1 | 0 | 1 | 0 | 0 |
| 39 | LOC619409 | 619409 | -8.82 | 5 | 0.181 | 22.675 | 0 | 0 | 0 | 0 | 0 | 0 | 0 |
| 40 | VCL | P18206 | -8.87 | 36 | 0.189 | 23.173 | 0 | 0 | 0 | 0 | 0 | 0 | 0 |
| 41 | LGMD1A | 6574 | -8.88 | 3 | 0.192 | 23.273 | 0 | 0 | 0 | 0 | 0 | 0 | 0 |
| 42 | SNTG1 | Q9NSN8 | -8.90 | 5 | 0.194 | 23.459 | 0 | 0 | 1 | 0 | 1 | 0 | 0 |
| 43 | EMD | P50402 | -8.94 | 12 | 0.201 | 23.864 | 0 | 0 | 0 | 0 | 0 | 0 | 0 |
| 44 | GNE | Q6QNY6 | -9.00 | 7 | 0.205 | 24.407 | 0 | 0 | 0 | 0 | 0 | 0 | 0 |
| 45 | MYOZ2 | Q9NPC6 | -9.03 | 7 | 0.209 | 24.632 | 0 | 0 | 0 | 0 | 0 | 0 | 0 |
| 46 | PGM5 | Q15124 | -9.04 | 3 | 0.212 | 24.733 | 0 | 0 | 1 | 0 | 0 | 0 | 0 |
| 47 | CASQ1 | P31415 | -9.05 | 5 | 0.213 | 24.892 | 0 | 0 | 0 | 0 | 0 | 0 | 0 |
| 48 | NR0B1 | P51843 | -9.06 | 18 | 0.218 | 25.047 | 0 | 0 | 0 | 0 | 0 | 0 | 0 |
| 49 | SYNC1 | Q9H7C4 | -9.08 | 4 | 0.219 | 25.066 | 0 | 0 | 0 | 0 | 0 | 0 | 0 |
| 50 | TTN | Q8WZ42 | -9.08 | 7 | 0.220 | 25.157 | 0 | 0 | 0 | 0 | 0 | 0 | 0 |
| 51 | DENR | O43583 | -9.12 | 3 | 0.228 | 25.497 | 0 | 0 | 0 | 0 | 0 | 0 | 0 |
| 52 | POMGNT1 | Q8WZA1 | -9.15 | 7 | 0.233 | 25.802 | 0 | 0 | 0 | 0 | 0 | 0 | 0 |
| 53 | RAPSN | Q13702 | -9.19 | 8 | 0.239 | 26.192 | 0 | 0 | 0 | 0 | 0 | 0 | 0 |
| 54 | MYOT | Q9UBF9 | -9.27 | 5 | 0.253 | 27.025 | 0 | 0 | 0 | 0 | 0 | 0 | 0 |
| 55 | GDF8 | O14793 | -9.28 | 5 | 0.254 | 27.080 | 0 | 0 | 0 | 0 | 0 | 0 | 0 |
| 56 | AIED | 351 | -9.30 | 2 | 0.256 | 27.193 | 0 | 0 | 0 | 0 | 0 | 0 | 0 |
| 57 | TRIM32 | Q13049 | -9.31 | 3 | 0.256 | 27.193 | 0 | 0 | 0 | 0 | 0 | 0 | 0 |
| 58 | MYH7 | P13533 | -9.36 | 18 | 0.265 | 27.894 | 0 | 0 | 0 | 0 | 0 | 0 | 0 |
| 59 | LAMB1 | P07942 | -9.36 | 6 | 0.266 | 27.898 | 0 | 0 | 0 | 0 | 0 | 0 | 0 |
| 60 | RP23 | 10277 | -9.41 | 6 | 0.274 | 28.242 | 0 | 0 | 0 | 0 | 0 | 0 | 0 |
| 61 | SNTG2 | Q05AH5 | -9.42 | 2 | 0.275 | 28.462 | 0 | 0 | 1 | 0 | 0 | 0 | 0 |
| 62 | ACTN3 | Q08043 | -9.46 | 5 | 0.284 | 28.783 | 0 | 0 | 0 | 0 | 0 | 0 | 0 |
| 63 | LMNA | P02545 | -9.46 | 17 | 0.285 | 28.814 | 0 | 0 | 0 | 0 | 0 | 0 | 0 |
| 64 | SPTBN4 | Q9H254 | -9.51 | 1 | 0.289 | 29.342 | 0 | 0 | 0 | 0 | 0 | 0 | 0 |
| 65 | OTC | P00480 | -9.55 | 8 | 0.298 | 29.590 | 0 | 0 | 0 | 0 | 0 | 0 | 0 |
| 66 | DTNBP1 | Q96EV8 | -9.56 | 5 | 0.299 | 29.616 | 0 | 0 | 0 | 0 | 0 | 0 | 0 |
| 67 | SNTB2 | Q13425 | -9.56 | 2 | 0.302 | 29.732 | 0 | 0 | 1 | 1 | 0 | 0 | 1 |
| 68 | LGMD1B | 6575 | -9.57 | 0 | 0.304 | 29.838 | 0 | 0 | 0 | 0 | 0 | 0 | 0 |
| 69 | SYNPO2 | Q9UMS6 | -9.57 | 3 | 0.307 | 29.862 | 0 | 0 | 0 | 0 | 0 | 0 | 0 |
| 70 | RPGR | Q4VX65 | -9.59 | 5 | 0.314 | 29.997 | 0 | 0 | 0 | 0 | 0 | 0 | 0 |
| 71 | SPTBN1 | Q01082 | -9.59 | 7 | 0.314 | 29.997 | 0 | 0 | 0 | 0 | 0 | 0 | 0 |
| 72 | GYPC | P04921 | -9.60 | 3 | 0.318 | 30.105 | 0 | 0 | 0 | 0 | 0 | 0 | 0 |
| 73 | TAZ | Q16635 | -9.63 | 8 | 0.329 | 30.387 | 0 | 0 | 0 | 0 | 0 | 0 | 0 |
| 74 | SNORD95 | 32757 | -9.63 | 3 | 0.329 | 30.387 | 0 | 0 | 0 | 0 | 0 | 0 | 0 |
| 75 | DMN | O15061 | -9.64 | 3 | 0.330 | 30.483 | 0 | 0 | 0 | 0 | 0 | 0 | 0 |
| 76 | SEPN1 | Q9NZV5 | -9.74 | 2 | 0.364 | 31.413 | 0 | 0 | 0 | 0 | 0 | 0 | 0 |
| 77 | GATM | P50440 | -9.76 | 2 | 0.370 | 31.678 | 0 | 0 | 0 | 0 | 0 | 0 | 0 |
| 78 | MTM1 | Q13496 | -9.78 | 5 | 0.372 | 31.823 | 0 | 0 | 0 | 0 | 0 | 0 | 0 |
| 79 | PLEC1 | Q15149 | -9.82 | 1 | 0.384 | 32.306 | 0 | 0 | 0 | 0 | 0 | 0 | 0 |
| 80 | NRG4 | Q0P6N4 | -9.82 | 1 | 0.387 | 32.363 | 0 | 0 | 0 | 0 | 0 | 0 | 0 |
| 81 | AAVS1 | 22 | -9.83 | 4 | 0.389 | 32.414 | 0 | 0 | 0 | 0 | 0 | 0 | 0 |
| 82 | MYOD1 | O75321 | -9.84 | 9 | 0.389 | 32.414 | 0 | 0 | 0 | 0 | 0 | 0 | 0 |
| 83 | FLNC | Q14315 | -9.87 | 3 | 0.398 | 32.802 | 0 | 0 | 0 | 0 | 0 | 0 | 0 |
| 84 | VAULTRC3 | 12656 | -9.88 | 1 | 0.400 | 32.846 | 0 | 0 | 0 | 0 | 0 | 0 | 0 |
| 85 | CFC1 | Q9GZR3 | -9.89 | 16 | 0.401 | 32.965 | 0 | 0 | 0 | 0 | 0 | 0 | 0 |
| 86 | IL1RAPL1 | Q7Z2K4 | -9.90 | 4 | 0.403 | 33.116 | 0 | 0 | 0 | 0 | 0 | 0 | 0 |
| 87 | DYNLT3 | P51808 | -9.91 | 3 | 0.406 | 33.239 | 0 | 0 | 0 | 0 | 0 | 0 | 0 |
| 88 | DTL | Q9NZJ0 | -9.93 | 2 | 0.411 | 33.417 | 0 | 0 | 0 | 0 | 0 | 0 | 0 |
| 89 | DMPK | Q09013 | -9.93 | 5 | 0.411 | 33.417 | 0 | 0 | 0 | 0 | 0 | 0 | 0 |
| 90 | MYOG | P15173 | -9.94 | 8 | 0.414 | 33.444 | 0 | 0 | 0 | 0 | 0 | 0 | 0 |
| 91 | DGKZ | Q13574 | -9.95 | 2 | 0.417 | 33.614 | 0 | 0 | 1 | 0 | 0 | 0 | 0 |
| 92 | SRRM2 | O60382 | -9.96 | 2 | 0.418 | 33.686 | 0 | 0 | 0 | 0 | 0 | 0 | 0 |
| 93 | SMN1 | Q16637 | -10.04 | 3 | 0.441 | 34.576 | 0 | 0 | 0 | 0 | 0 | 0 | 0 |
| 94 | MYL2 | P10916 | -10.05 | 2 | 0.445 | 34.750 | 0 | 0 | 0 | 0 | 0 | 0 | 0 |
| 95 | MYLPF | Q6IB41 | -10.09 | 2 | 0.457 | 35.062 | 0 | 0 | 0 | 0 | 0 | 0 | 0 |
| 96 | PVALB | P02144 | -10.10 | 22 | 0.464 | 35.210 | 0 | 0 | 0 | 0 | 0 | 0 | 0 |
| 97 | COL6A1 | P12109 | -10.14 | 2 | 0.473 | 35.566 | 0 | 0 | 0 | 0 | 0 | 0 | 0 |
| 98 | MYH7 | P12883 | -10.14 | 2 | 0.474 | 35.583 | 0 | 0 | 0 | 0 | 0 | 0 | 0 |
| 99 | CAPN8 | 1485 | -10.14 | 1 | 0.476 | 35.607 | 0 | 0 | 0 | 0 | 0 | 0 | 0 |
| 100 | MEAX | 6987 | -10.15 | 2 | 0.477 | 35.638 | 0 | 0 | 0 | 0 | 0 | 0 | 0 |
| 101 | POMT2 | Q59GJ5 | -10.15 | 0 | 0.479 | 35.702 | 0 | 0 | 0 | 0 | 0 | 0 | 0 |
| 102 | AGRN | O00468 | -10.18 | 3 | 0.483 | 35.963 | 0 | 0 | 0 | 0 | 0 | 0 | 0 |
| 103 | DNPEP | Q9HAC6 | -10.18 | 2 | 0.484 | 35.967 | 0 | 0 | 0 | 0 | 0 | 0 | 0 |
| 104 | XIC | 12809 | -10.19 | 0 | 0.491 | 36.045 | 0 | 0 | 0 | 0 | 0 | 0 | 0 |
| 105 | PDLIM3 | Q53GG5 | -10.20 | 2 | 0.499 | 36.198 | 0 | 0 | 0 | 0 | 0 | 0 | 0 |
| 106 | COL6A2 | P12110 | -10.21 | 1 | 0.500 | 36.268 | 0 | 0 | 0 | 0 | 0 | 0 | 0 |
| 107 | GAA | P10253 | -10.21 | 7 | 0.501 | 36.300 | 0 | 0 | 0 | 0 | 0 | 0 | 0 |
| 108 | LAMA1 | P25391 | -10.26 | 0 | 0.520 | 36.811 | 0 | 0 | 0 | 0 | 0 | 0 | 0 |
| 109 | MYF6 | P23409 | -10.27 | 2 | 0.524 | 36.845 | 0 | 0 | 0 | 0 | 0 | 0 | 0 |
| 110 | CHRNG | P07510 | -10.29 | 1 | 0.531 | 37.012 | 0 | 0 | 0 | 0 | 0 | 0 | 0 |
| 111 | SPTA1 | O60686 | -10.30 | 2 | 0.535 | 37.144 | 0 | 0 | 0 | 0 | 0 | 0 | 0 |
| 112 | CSRP3 | P50461 | -10.30 | 3 | 0.542 | 37.216 | 0 | 0 | 0 | 0 | 0 | 0 | 0 |
| 113 | EPB41 | P11171 | -10.31 | 4 | 0.548 | 37.322 | 0 | 0 | 0 | 0 | 0 | 0 | 0 |
| 114 | PBDX | P55808 | -10.32 | 1 | 0.548 | 37.322 | 0 | 0 | 0 | 0 | 0 | 0 | 0 |
| 115 | LAMB2 | P55268 | -10.32 | 1 | 0.549 | 37.398 | 0 | 0 | 0 | 0 | 0 | 0 | 0 |
| 116 | WDM | 50988 | -10.34 | 1 | 0.561 | 37.627 | 0 | 0 | 0 | 0 | 0 | 0 | 0 |
| 117 | HHG | 4902 | -10.35 | 1 | 0.563 | 37.680 | 0 | 0 | 0 | 0 | 0 | 0 | 0 |
| 118 | RPS4Y1 | P22090 | -10.35 | 2 | 0.563 | 37.680 | 0 | 0 | 0 | 0 | 0 | 0 | 0 |
| 119 | ITGA7 | Q13683 | -10.35 | 1 | 0.563 | 37.680 | 0 | 0 | 0 | 0 | 0 | 0 | 0 |
| 120 | TNNT2 | P45379 | -10.37 | 4 | 0.574 | 37.877 | 0 | 0 | 0 | 0 | 0 | 0 | 0 |
| 121 | CMD1B | 2102 | -10.37 | 2 | 0.574 | 37.877 | 0 | 0 | 0 | 0 | 0 | 0 | 0 |
| 122 | FOSL2 | P15408 | -10.38 | 1 | 0.578 | 38.028 | 0 | 0 | 0 | 0 | 0 | 0 | 0 |
| 123 | SFRS2 | Q01130 | -10.39 | 3 | 0.582 | 38.131 | 0 | 0 | 0 | 0 | 0 | 0 | 0 |
| 124 | MIB2 | Q0JSM5 | -10.40 | 1 | 0.590 | 38.257 | 0 | 0 | 0 | 0 | 0 | 0 | 0 |
| 125 | MSRB2 | Q9Y3D2 | -10.40 | 1 | 0.590 | 38.257 | 0 | 0 | 0 | 0 | 0 | 0 | 0 |
| 126 | DNM1L | O00429 | -10.40 | 1 | 0.590 | 38.257 | 0 | 0 | 0 | 0 | 0 | 0 | 0 |
| 127 | XKR1 | P51811 | -10.41 | 2 | 0.593 | 38.307 | 0 | 0 | 0 | 0 | 0 | 0 | 0 |
| 128 | XIST | 12810 | -10.42 | 0 | 0.605 | 38.416 | 0 | 0 | 0 | 0 | 0 | 0 | 0 |
| 129 | TPM1 | O15513 | -10.42 | 5 | 0.606 | 38.439 | 0 | 0 | 0 | 0 | 0 | 0 | 0 |
| 130 | COL6A3 | P12111 | -10.42 | 1 | 0.610 | 38.522 | 0 | 0 | 0 | 0 | 0 | 0 | 0 |
| 131 | SUCLG1 | P53597 | -10.42 | 1 | 0.613 | 38.547 | 0 | 0 | 0 | 0 | 0 | 0 | 0 |
| 132 | NRG3 | P56975 | -10.43 | 1 | 0.614 | 38.587 | 0 | 0 | 0 | 0 | 0 | 0 | 0 |
| 133 | PPP1R10 | Q96QC0 | -10.43 | 1 | 0.614 | 38.587 | 0 | 0 | 0 | 0 | 0 | 0 | 0 |
| 134 | RNPS1 | Q15287 | -10.44 | 2 | 0.623 | 38.738 | 0 | 0 | 0 | 0 | 0 | 0 | 0 |
| 135 | MYEF2 | Q9P2K5 | -10.46 | 2 | 0.632 | 38.880 | 0 | 0 | 0 | 0 | 0 | 0 | 0 |
| 136 | GAMT | Q14353 | -10.48 | 1 | 0.646 | 39.130 | 0 | 0 | 0 | 0 | 0 | 0 | 0 |
| 137 | TNNC1 | P63316 | -10.49 | 3 | 0.650 | 39.232 | 0 | 0 | 0 | 0 | 0 | 0 | 0 |
| 138 | RP2 | O75695 | -10.51 | 3 | 0.661 | 39.446 | 0 | 0 | 0 | 0 | 0 | 0 | 0 |
| 139 | MYL6 | P60660 | -10.51 | 1 | 0.663 | 39.469 | 0 | 0 | 0 | 0 | 0 | 0 | 0 |
| 140 | CTSH | P09668 | -10.51 | 2 | 0.664 | 39.480 | 0 | 0 | 0 | 0 | 0 | 0 | 0 |
| 141 | CXADR | P78310 | -10.53 | 4 | 0.681 | 39.609 | 0 | 0 | 0 | 0 | 0 | 0 | 0 |
| 142 | ELOVL4 | Q9GZR5 | -10.53 | 1 | 0.682 | 39.635 | 0 | 0 | 0 | 0 | 0 | 0 | 0 |
| 143 | MYF5 | P13349 | -10.57 | 3 | 0.703 | 40.025 | 0 | 0 | 0 | 0 | 0 | 0 | 0 |
| 144 | FBXO32 | Q969P5 | -10.59 | 1 | 0.716 | 40.275 | 0 | 0 | 0 | 0 | 0 | 0 | 0 |
| 145 | PRX | Q9BXM0 | -10.60 | 2 | 0.716 | 40.275 | 0 | 0 | 0 | 0 | 0 | 0 | 0 |
| 146 | BLOC1S1 | P78537 | -10.60 | 1 | 0.718 | 40.311 | 0 | 0 | 0 | 0 | 0 | 0 | 0 |
| 147 | MUSK | O15146 | -10.60 | 1 | 0.718 | 40.311 | 0 | 0 | 0 | 0 | 0 | 0 | 0 |
| 148 | SMN2 | Q16637 | -10.62 | 1 | 0.731 | 40.491 | 0 | 0 | 0 | 0 | 0 | 0 | 0 |
| 149 | IS2 | 282552 | -10.62 | 0 | 0.734 | 40.516 | 0 | 0 | 0 | 0 | 0 | 0 | 0 |
| 150 | DM1 | 2923 | -10.62 | 2 | 0.735 | 40.521 | 0 | 0 | 0 | 0 | 0 | 0 | 0 |
| 151 | DYNLL1 | P63167 | -10.63 | 1 | 0.737 | 40.567 | 0 | 0 | 0 | 0 | 0 | 0 | 0 |
| 152 | PDAP1 | Q13442 | -10.63 | 1 | 0.737 | 40.567 | 0 | 0 | 0 | 0 | 0 | 0 | 0 |
| 153 | INVS | Q5JS85 | -10.65 | 3 | 0.748 | 40.760 | 0 | 0 | 0 | 0 | 0 | 0 | 0 |
| 154 | PABPN1 | Q86U42 | -10.66 | 1 | 0.760 | 40.877 | 0 | 0 | 0 | 0 | 0 | 0 | 0 |
| 155 | NOS1AP | O75052 | -10.67 | 1 | 0.762 | 40.915 | 0 | 0 | 0 | 0 | 0 | 0 | 0 |
| 156 | KCNJ10 | P78508 | -10.67 | 3 | 0.765 | 40.985 | 0 | 0 | 0 | 0 | 0 | 0 | 0 |
| 157 | TCTA | P57738 | -10.68 | 0 | 0.767 | 41.034 | 0 | 0 | 0 | 0 | 0 | 0 | 0 |
| 158 | ACTA1 | P68133 | -10.68 | 2 | 0.769 | 41.074 | 0 | 0 | 1 | 0 | 0 | 0 | 0 |
| 159 | CACNA1I | Q9P0X4 | -10.68 | 1 | 0.776 | 41.155 | 0 | 0 | 0 | 0 | 0 | 0 | 0 |
| 160 | MST4 | Q8NC04 | -10.71 | 1 | 0.786 | 41.424 | 0 | 0 | 0 | 0 | 0 | 0 | 0 |
| 161 | KFSD | 6313 | -10.72 | 2 | 0.786 | 41.424 | 0 | 0 | 0 | 0 | 0 | 0 | 0 |
| 162 | IGFBP5 | P24593 | -10.72 | 1 | 0.789 | 41.494 | 0 | 0 | 0 | 0 | 0 | 0 | 0 |
| 163 | DST | O94833 | -10.75 | 7 | 0.812 | 41.721 | 0 | 0 | 0 | 0 | 0 | 0 | 0 |
| 164 | FRG1 | Q14331 | -10.76 | 0 | 0.820 | 41.913 | 0 | 0 | 0 | 0 | 0 | 0 | 0 |
| 165 | CD5L | O43866 | -10.77 | 0 | 0.823 | 41.983 | 0 | 0 | 0 | 0 | 0 | 0 | 0 |
| 166 | ITPR1 | Q14643 | -10.79 | 1 | 0.830 | 42.123 | 0 | 0 | 0 | 0 | 0 | 0 | 0 |
| 167 | PARVB | Q9HBI1 | -10.80 | 0 | 0.838 | 42.202 | 0 | 0 | 0 | 0 | 0 | 0 | 0 |
| 168 | RIMS1 | Q5SZK2 | -10.80 | 1 | 0.838 | 42.202 | 0 | 0 | 0 | 0 | 0 | 0 | 0 |
| 169 | GAS2 | O43903 | -10.80 | 3 | 0.845 | 42.293 | 0 | 0 | 0 | 0 | 0 | 0 | 0 |
| 170 | WAS | P42768 | -10.80 | 238 | 0.846 | 42.308 | 0 | 0 | 0 | 0 | 0 | 0 | 0 |
| 171 | CDH15 | P55291 | -10.81 | 2 | 0.849 | 42.363 | 0 | 0 | 0 | 0 | 0 | 0 | 0 |
| 172 | ACTC1 | P68032 | -10.82 | 1 | 0.850 | 42.429 | 0 | 0 | 1 | 0 | 0 | 0 | 0 |
| 173 | MLS | 7145 | -10.82 | 1 | 0.850 | 42.429 | 0 | 0 | 0 | 0 | 0 | 0 | 0 |
| 174 | CACNA1S | Q13698 | -10.82 | 3 | 0.851 | 42.507 | 0 | 0 | 0 | 0 | 0 | 0 | 0 |
| 175 | ERF | P50548 | -10.83 | 1 | 0.856 | 42.558 | 0 | 0 | 0 | 0 | 0 | 0 | 0 |
| 176 | SFRS1 | Q07955 | -10.84 | 1 | 0.865 | 42.672 | 0 | 0 | 0 | 0 | 0 | 0 | 0 |
| 177 | DCTN3 | O75935 | -10.87 | 1 | 0.894 | 42.976 | 0 | 0 | 0 | 0 | 0 | 0 | 0 |
| 178 | DDX3Y | O15523 | -10.87 | 1 | 0.894 | 42.976 | 0 | 0 | 0 | 0 | 0 | 0 | 0 |
| 179 | SFRS5 | Q13243 | -10.87 | 1 | 0.896 | 43.018 | 0 | 0 | 0 | 0 | 0 | 0 | 0 |
| 180 | ALG3 | Q92685 | -10.87 | 109 | 0.896 | 43.018 | 0 | 0 | 0 | 0 | 0 | 0 | 0 |
| 181 | RYR1 | O75591 | -10.88 | 1 | 0.908 | 43.141 | 0 | 0 | 0 | 0 | 0 | 0 | 0 |
| 182 | GAS2L1 | Q99501 | -10.90 | 1 | 0.919 | 43.308 | 0 | 0 | 0 | 0 | 0 | 0 | 0 |
| 183 | COL4A5 | P29400 | -10.90 | 0 | 0.919 | 43.308 | 0 | 0 | 0 | 0 | 0 | 0 | 0 |
| 184 | PTBP2 | O95652 | -10.91 | 0 | 0.926 | 43.393 | 0 | 0 | 0 | 0 | 0 | 0 | 0 |
| 185 | MYH6 | P13533 | -10.91 | 4 | 0.928 | 43.444 | 0 | 0 | 0 | 0 | 0 | 0 | 0 |
| 186 | IGFBP4 | P22692 | -10.92 | 3 | 0.933 | 43.582 | 0 | 0 | 0 | 0 | 0 | 0 | 0 |
| 187 | SYNE1 | Q5JV23 | -10.93 | 1 | 0.934 | 43.620 | 0 | 0 | 0 | 0 | 0 | 0 | 0 |
| 188 | ZNF91 | Q05481 | -10.93 | 1 | 0.939 | 43.637 | 0 | 0 | 0 | 0 | 0 | 0 | 0 |
| 189 | SP1 | P08047 | -10.93 | 7 | 0.940 | 43.669 | 0 | 0 | 0 | 0 | 0 | 0 | 0 |
| 190 | PTPN22 | Q5TBC0 | -10.93 | 1 | 0.941 | 43.671 | 0 | 0 | 0 | 0 | 0 | 0 | 0 |
| 191 | LOC619511 | 619511 | -10.94 | 1 | 0.943 | 43.701 | 0 | 0 | 0 | 0 | 0 | 0 | 0 |
| 192 | EIF4EBP1 | Q13541 | -10.95 | 3 | 0.953 | 43.838 | 0 | 0 | 0 | 0 | 0 | 0 | 0 |
| 193 | MYOZ1 | Q9NP98 | -10.97 | 0 | 0.977 | 44.029 | 0 | 0 | 0 | 0 | 0 | 0 | 0 |
| 194 | BSN | Q2NLD3 | -10.98 | 0 | 0.988 | 44.167 | 0 | 0 | 0 | 0 | 0 | 0 | 0 |
| 195 | FBXO11 | Q86XK2 | -10.99 | 1 | 0.999 | 44.248 | 0 | 0 | 0 | 0 | 0 | 0 | 0 |
| 196 | ZBTB20 | Q9HC78 | -10.99 | 1 | 0.999 | 44.248 | 0 | 0 | 0 | 0 | 0 | 0 | 0 |
